# Folate-Functionalized ROS-Scavenging Covalent Organic Framework for Oral Targeted Delivery of Ferulic Acid in Ulcerative Colitis

**DOI:** 10.3390/pharmaceutics17101263

**Published:** 2025-09-26

**Authors:** Jin Xue, Zifan Qiao, Shiyu Huang, Mubarak G. Bello, Lihua Chen

**Affiliations:** 1 Formula-Pattern Research Center, Jiangxi University of Chinese Medicine, No. 1688, Meiling Road, Nanchang 330004, China; xuejin@jxutcm.edu.cn; 2 Key Laboratory of Modern Preparation of TCM, Ministry of Education, Jiangxi University of Chinese Medicine, No. 1688, Meiling Road, Nanchang 330004, China; qzf4901@163.com; 3 School of Pharmacy, Jiangxi Science and Technology Normal University, Nanchang 330013, China; 2023020049@jxstnu.edu.cn; 4 Department of Pharmaceutics and Industrial Pharmacy, Kaduna State University, Kaduna PMB 2339, Nigeria

**Keywords:** cyclodextrin, covalent organic framework, ROS-scavenging, ulcerative colitis, ferulic acid, targeted delivery

## Abstract

**Background/Objectives:** Ulcerative colitis (UC) involves chronic colon inflammation and oxidative stress. Treating UC is challenging due to systemic drug side effects and poor targeted delivery. Nanocarriers responsive to the UC microenvironment, particularly elevated reactive oxygen species (ROS), could overcome these limitations. This study developed an oral delivery system for ROS-triggered drug release and active targeting. Using ferulic acid (FER), a system was designed to enhance site-specific accumulation and therapeutic efficacy against colitis. **Methods:** A ROS-sensitive covalent organic framework (COF) was synthesized from γ-cyclodextrin and functionalized with folic acid (FA) to create a carrier (COF-FA) designed for potential active targeting. This carrier was loaded with FER to form FER@COF-FA. The system was characterized (SEM, FTIR, TGA), and its ROS scavenging and sustained drug release profiles were confirmed in vitro. Biocompatibility was evaluated in cell lines, and therapeutic efficacy was tested in a DSS-induced murine colitis model. **Results:** The synthesized FER@COF-FA demonstrated high drug loading, potent ROS-scavenging capability, and a sustained drug release profile. It showed excellent biocompatibility and, in the murine model, significantly outperformed free FER. Treatment alleviated disease severity, prevented colon shortening, restored healthy tissue histology, and rebalanced pro- and anti-inflammatory cytokines. **Conclusions:** The FER@COF-FA system represents a highly promising therapeutic strategy for UC. Its superior efficacy is attributed to a synergistic multi-mechanism approach, combining sustained release, ROS-responsive drug delivery, intrinsic antioxidant activity, and potential folate receptor-mediated targeting, which collectively enhance site-specific accumulation and therapeutic outcomes in the inflammatory colon microenvironment.

## 1. Introduction

Ulcerative colitis is a chronic, relapsing inflammatory bowel disease characterized by a dysregulated immune response and persistent mucosal inflammation [1,2,3]. Its rising global prevalence poses a significant clinical challenge, compounded by the limitations of current therapies [4]. Mainstay treatments, such as corticosteroids and immunosuppressants, often cause broad systemic side effects, including hyperglycemia, hypertension, increased susceptibility to infections, and bone loss with corticosteroids, as well as elevated risks of hepatotoxicity, nephrotoxicity, and malignancy with long-term use of immunosuppressants [5,6]. Biologic agents, while more targeted, face issues with primary non-response and loss of efficacy over time [7]. The therapeutic goals in UC management are to induce and maintain clinical and endoscopic remission, prevent disease complications, improve quality of life, and minimize treatment-related adverse effects [5]. This highlights a critical unmet need for novel strategies that can enhance drug delivery specifically to the site of inflammation, thereby maximizing therapeutic efficacy while minimizing off-target toxicity.

A promising strategy involves engineering a drug delivery system with multi-functional capabilities tailored to the pathological microenvironment of UC. The inflamed colon is characterized by two key features: massive infiltration of activated macrophages and a state of intense oxidative stress marked by excessive reactive oxygen species [8,9]. This pathological landscape provides dual targets for smart drug delivery through a cellular target via receptors overexpressed on macrophages, and a chemical target via the abundant ROS.

To exploit the cellular target, the folate receptor represents a compelling target, as it is minimally expressed under physiological conditions but markedly upregulated on activated macrophages in inflamed tissues [10,11,12]. FA conjugation offers a well-established route to target these cells [13,14,15]. To exploit the chemical target, a ROS-sensitive carrier can be designed to enhance site-specific drug release and prolong therapeutic action at inflamed tissues, thereby overcoming the current challenges of poor accumulation and rapid clearance in ulcerative colitis treatment [16,17,18]. Furthermore, to address the poor pharmacokinetic profile of many potent therapeutic compounds, a system capable of providing sustained release is essential for maintaining effective local drug concentrations [19,20,21,22].

Ferulic acid, a natural polyphenol with proven antioxidant and anti-inflammatory properties, is an ideal drug candidate for UC treatment [23,24,25]. However, its clinical translation is severely limited by poor aqueous solubility, low bioavailability, and rapid systemic clearance [26,27]. To overcome these hurdles, a covalent cyclodextrin organic framework (COF) represents a versatile encapsulation platform, offering high porosity, excellent stability, and the ability to be engineered for specific functions [28,29,30].

This study presents the rational design and evaluation of a multi-functional carrier system that synergistically combines three therapeutic strategies into a single platform. A ROS-sensitive, cyclodextrin-based COF was synthesized and functionalized with folic acid (COF-FA) to create a carrier designed for: (1) sustained release of its payload from the porous COF matrix; (2) intrinsic ROS-scavenging activity through the hydrolysis of its oxalate bonds in the inflammatory microenvironment; and (3) potential active targeting of activated macrophages exploiting folate-mediated uptake. This carrier was subsequently loaded with ferulic acid to form FER@COF-FA.

The system was comprehensively characterized, confirming its successful synthesis, ROS-scavenging capability, and sustained drug release profile in vitro. The therapeutic efficacy was then rigorously evaluated in a murine model of DSS-induced colitis. Results demonstrate that FER@COF-FA significantly outperforms free FER, providing superior alleviation of disease activity, prevention of colon shortening, normalization of systemic inflammation, and restoration of healthy cytokine balance. The significantly enhanced outcomes align with the design hypothesis, suggesting successful receptor-mediated targeting and highlighting the advantage of a multi-mechanistic approach. This work establishes FER@COF-FA as a highly promising and sophisticated platform for the targeted treatment of inflammatory bowel diseases.

## 2. Method

### 2.1. Materials

γ-Cyclodextrin (γ-CD) was sourced from Maxdragon Biochem Ltd. (Guangzhou, China). Analytical-grade reagents including potassium hydroxide (KOH), polyethylene glycol 20000 (PEG 20000), monopotassium phosphate, ethanol, and methanol were purchased from Sinopharm Chemical Reagent Co., Ltd. (Shanghai, China). Oxalyl chloride (98%) was purchased from Aladdin Biochemical Technology Co., Ltd. (Shanghai, China), anhydrous dichloromethane (Batch: LC60X04) from Beijing InnoChem Science & Technology Co., Ltd. (Beijing, China), and triethylamine (Batch: P3066131) from Shanghai Titan Scientific Co., Ltd. (Shanghai, China). Additionally sourced were anhydrous dimethyl sulfoxide (99.9%, Beijing InnoChem Science & Technology), anhydrous ethanol (99.9%, Shanghai Titan Scientific), folic acid (Batch: P2743040, Shanghai Titan Scientific), and ferulic acid (Batch: F2428189, Aladdin Biochemical Technology). N,N′-dicyclohexylcarbodiimide (99.0%) was procured from Shanghai Macklin Biochemical Co., Ltd. (Shanghai, China). Dulbecco’s Modified Eagle Medium (DMEM), Fetal bovine serum (FBS), and CCK-8 were procured from Solarbio Science and Technology Biotechnology Co., Ltd. (Beijing, China). All chemicals were commercially available and used without further purification.

Human epithelial colorectal adenocarcinoma cell line (Caco2) was purchased from Dalian Meilun Biotechnology Co., Ltd. (Dalian, China) (Product code: PWE-HU027; Cell batch number: 20210823A2) and murine macrophage cell line (RAW 264.7) were obtained from Suzhou Haixing Biotechnology Co., Ltd. (Suzhou, China) (Cell Batch Number: 240517X201). As part of our quality control, all cell lines were confirmed to be free of mycoplasma contamination and were used at a low passage number following receipt from the supplier.

### 2.2. Micro γ-CD-MOF Preparation

CD-MOF was prepared using a modified hydrothermal method according to the reported literature [31]. Microscale γ-CD-MOF crystals were synthesized from γ-CD (32.5 g, 1 mmol) and KOH (11.2 g, 8 mmol) dissolved in 1 L ultra-pure water. Methanol (600 mL) was added and the reaction maintained for 20 min until a clear solution was formed, followed by addition of PEG 20000 (12.8 g) as a stabilizer and the reaction was allowed to continue for 10 min. The clear solution obtained was collected and cooled at 15 °C overnight to precipitate cubic crystals, which were then thoroughly washed with ethanol and vacuum-dried at 45 °C for 12 h.

### 2.3. Synthesis of ROS-Sensitive Crosslinked Cyclodextrin Framework

COF was synthesized using CD-MOF powder and oxalyl chloride (OC) as a crosslinker [28]. Briefly, CD-MOF (1.5 g, 0.46 mmol) was weighed into a single-neck round-bottom flask, followed by the addition of anhydrous dichloromethane (DCM, 15 mL). After ultrasonic agitation, the mixture was stirred in an ice bath. Additionally, triethylamine (TEA, 0.5 mL, 3.6 mmol) was introduced as a catalyst, and the flask was evacuated three times to establish a nitrogen atmosphere. Subsequently, OC (0.55 mL, 6.4 mmol) dissolved in 2.5 mL of anhydrous dichloromethane was slowly injected into the reaction system via a rubber tube connected to the flask’s stopcock to prevent air ingress. The reaction proceeded overnight (12 h) under nitrogen at 25 °C. The product obtained was washed twice with a series of absolute ethanol, ethanol-water, and pure water to eliminate the unreacted OC. Finally, the centrifuged product was lyophilized using SCIENTZ-12N (Ningbo Scientz Biotechnology Co., Ltd., Ningbo, China) at −40 °C and 0.003 Pa for 24 h to obtain the final COF product.

### 2.4. Ferulic Acid Incorporation

Briefly, FER (400 mg) was dissolved in 5 mL of anhydrous ethanol via ultrasonication. COF (100 mg) was added, and the mixture was magnetically stirred at 37 °C (400 rpm) for 6 h in the dark. The obtained product was centrifuged to remove the supernatant, washed three times with anhydrous ethanol to eliminate surface-adsorbed FER, and vacuum-dried at 45 °C for 5 h to obtain FER@COF particles.

To determine the drug loading percent (%), FER@COF (5 mg) was dissolved in ultrapure water, diluted to 10 mL, and allowed to stand until complete dissolution. A 1 mL aliquot was diluted to 10 mL, sonicated for 5 min, and analyzed by UV–Vis spectrophotometry to determine FER loading at 280 nm wavelength.

### 2.5. Folic Acid Functionalized ROS-Sensitive COF

FA (0.51 g, 1.15 mmol) was dissolved in 25 mL of anhydrous dimethyl sulfoxide (DMSO) in a round-bottom flask with the aid of sonication. N,N′-Dicyclohexylcarbodiimide (0.8 g, 3.88 mmol) and 4-dimethylaminopyridine (0.5 g, 4.1 mmol) were then added to the FA/DMSO solution and stirred for 4 h to activate carboxyl groups [32]. COF and FER@COF powder were then introduced to the activated FA mixture, and the reaction was carried out in the dark for 24 h. The product precipitate was collected by centrifugation, washed three times with anhydrous ethanol and water. The obtained product was then lyophilized (SCIENTZ-12N) at −40 °C and 0.003 Pa for 24 h to yield COF-FA and FER@COF-FA powder.

Measurement of FA grafting efficiency: The grafting efficiency of FA was determined by HPLC analysis of the unreacted FA remaining in the supernatant and washings after the coupling reaction. The combined filtrates were collected, diluted to a known volume, filtered (0.22 µm), and analyzed using an Agilent 1260 Infinity HPLC system (Agilent Technologies, Santa clara, CA, USA) with an ACE EXCEL 5 C18 column (250 × 4.6 mm, 5 µm) at 25 °C. The mobile phase consisted of phosphate buffer (pH 4.0) and methanol (99:1, *v*/*v*) at a flow rate of 1.2 mL/min, with detection at 254 nm [33].

The FA grafting efficiency was calculated according to the equation:

FA grafting efficiency (%) = mFA,initial − mFA,unreacted/mCOF-FAproduct × 100%(1)

where mFA,initial is the initial mass of FA added, mFA,unreacted is the mass of FA determined by HPLC in the supernatant and washings, and mCOF-FAproduct is the mass of dried COF-FA obtained. All measurements were performed in triplicate, and results are reported as mean ± SD.

### 2.6. Physicochemical Characterization

The morphological features of COF, COF-FA, and FER@COF-FA were examined using SEM (TESCAN MIRA LMS, Brno, Czech Republic). The analysis was operated at a beam voltage of 15 kV and equipped with an energy-dispersive X-ray spectroscopy (EDX) detector. Prior to measurement, the samples were mounted onto a steel stage using double-sided adhesive tape and sputter-coated with a thin gold film to enhance conductivity and prevent charging. The crystallinity of the samples before and after crosslinking was investigated using powder X-ray diffractometry (PXRD). FTIR spectra were acquired over 4000–400 cm^−1^ at 1 cm^−1^ resolution. Briefly, samples of COF, FER, FA, COF-FA, and FER@COF-FA (1:100 mass ratio to KBr) were homogenized in an agate mortar and pressed into pellets. The thermal stability of FER, COF-FA, and FER@COF-FA was analyzed using a TG-DSC instrument (Netzsch STA 449 F3, WittelsbacherstraBe, Germany) from 30 °C to 600 °C at 20 °C/min under nitrogen.

### 2.7. In Vitro Antioxidant Capability of COF-FA

The H_2_O_2_ scavenging ability was evaluated by measuring the residual H_2_O_2_ concentration after incubation with COF-FA, as reported in the literature [28,34]. Various concentrations of COF-FA (20, 40, or 80 mg) was added to 10 mL of 150 μM H_2_O_2_ in amber vials. Control samples contained H_2_O_2_ without COF-FA. All samples were incubated at 37 °C with mechanical stirring (100 rpm). At 1, 2, 4, 8, 12, and 24 h, 300 μL aliquots were centrifuged (12,000 rpm, 5 min), and supernatants were analyzed using a hydrogen peroxide assay kit. Experiments were performed in triplicate. Additional tests were conducted using 250 μM H_2_O_2_ under identical conditions.

### 2.8. In Vitro Release Study

The release profile of FER was evaluated utilizing a dialysis bag technique. A pre-hydrated cellulose membrane with a molecular weight cutoff (MWCO) of 3000 kDa was loaded with 5.0 mL of the sample (aqueous FER or FER@COF-FA), which was then immersed in a 40 mL PBS receptor solution maintained at 37 °C under constant agitation. At predetermined intervals, 1.0 mL aliquots were withdrawn, and the FER concentration was quantified via HPLC. Following each sampling event, an equal volume of fresh PBS was replenished to ensure a consistent receptor volume and osmolality.

The concentration of FER was quantitatively determined using HPLC on an Agilent 1260 Infinity system. Chromatographic separation was achieved using a Kinetex^®^ 5 μm XB-C18 column (150 mm × 4.6 mm, 5 μm particle size; Phenomenex, Torrance, CA, USA) maintained at 40 °C. The mobile phase consisted of an aqueous component containing 0.1% (*v*/*v*) phosphoric acid and methanol, delivered in an isocratic ratio of 60:40 (aqueous–methanol) at a flow rate of 1.0 mL/min. The injection volume was 20 μL, and detection was performed using a ultraviolet-visible (UV-Vis) detector set at a wavelength of 308 nm, corresponding to the characteristic absorption maximum of the analyte [35].

### 2.9. Cell Viability Assay

RAW264.7 and Caco2 cells were seeded in 96-well plates at 10,000 cells/well and incubated for 24 h (37 °C, 95% air, 5% CO_2_). Cells were treated with COF, COF-FA, and FER@COF-FA at various concentrations. Subsequently, 100 μL of CCK-8 solution (0.5 mg/mL) was added to each well. After 2 h incubation, absorbance at 450 nm was measured using a microplate reader (GENios Tecan, Seestrasse 103, Männedorf, Switzerland). Six replicates per concentration and three independent experiments were performed. Untreated cells and culture medium served as the control and blank, respectively.

### 2.10. In Vivo Study

#### 2.10.1. Animal Grouping and Study Design

Thirty male Kunming (KM) mice (6–8 weeks, 33.0 ± 2.0 g, specific pathogen-free [SPF] grade) were obtained from the Experimental Animal Science Center of Jiangxi University of Chinese Medicine (License No.: SCXK(Gan)2023-0001). All procedures complied with the Institutional Animal Care and Use Committee guidelines (JZLLSC-20240590). Thirty SPF-grade male KM mice were randomly divided into five groups (n = 6): Control group, DSS group, FER group (60 mg/kg body weight), COF-FA group (400 mg/kg), and FER@COF-FA group (400 mg/kg, containing 60 mg/kg FER).

The mice were housed at 22 ± 2 °C and 50 ± 5% humidity under a 12-h light/dark cycle. After a one-week acclimatization period with free access to distilled water and a standard diet, UC was induced in all groups except the control group by administering 3.0% (*w*/*v*) dextran sulfate sodium (DSS) in the drinking water for 7 days, in accordance with well-established protocols. The DSS group received 3% DSS ad libitum as drinking water and did not receive any treatment. Treatments were administered daily via oral gavage from day 3 to day 7. All formulations, including free FER, COF-FA, and FER@COF-FA, were prepared as homogeneous suspensions in purified water and administered at a fixed volume of 0.3 mL per mouse. Each suspension was vortexed thoroughly and briefly sonicated immediately before gavage to ensure uniform dispersion and dosing accuracy. On the eighth day, mice were anesthetized. Blood was collected via retro-orbital puncture, after which the animals were euthanized. Subsequently, the colon length and spleen weight were measured. A segment of the distal colon (approximately 1 cm) was collected and stained with hematoxylin and eosin (H&E) for histopathological analysis.

#### 2.10.2. Disease Activity Index (DAI) Evaluation

During DSS treatment, body weight, stool characteristics and blood in the stool were recorded every day and scored from 0 to 4 points: weight loss (0: 0–1%; 1: 1–5%; 2: 5–10%; 3: 10–15%; 4: >15%, percentage refers to mass fraction); stool consistency (0: normal; 2: loose stool; 4: diarrhea); and stool bleeding (0: normal; 2: blood; 4: total bleeding) [36]. DAI was calculated as the average score.

#### 2.10.3. Determination of Colon Length and Spleen Index

Body weight was recorded before euthanasia. The colon was excised from the cecocolonic junction to the proximal rectum and measured. Spleens were weighed to calculate the spleen index:

Spleen index (%) = [Spleen weight (g)/Final body weight (g)] × 100%(2)

#### 2.10.4. Analysis of Colon Tissue Pathology Sections

The end of the colon was taken at 0.5 cm. After being fixed in 4% paraformaldehyde solution for 24 h, the tissue was dehydrated using a gradient of ethanol, anhydrous ethanol, and xylene, embedded in paraffin, and then sliced into 5-μm-thick sections using a slicer. The slices were placed in an oven at 70 °C and cooled to room temperature. The paraffin slices were dewaxed in xylene I, xylene II, anhydrous ethanol I, anhydrous ethanol II, and 75% alcohol in sequence. The slices were washed with water and stained with hematoxylin. After differentiation in 0.65% ammonia water and eosin counterstaining, slides were dehydrated, mounted with neutral balsam, and imaged by microscopy.

#### 2.10.5. Serum Inflammatory Cytokine Measurement

Serum samples were isolated by centrifugation at 4000 rpm for 10 min at 4 °C. The concentrations of interleukin-6 (IL-6, Catalog No. 2501M06), tumor necrosis factor-α (TNF-α, Catalog No. 2501M19), and interleukin-10 (IL-10, Catalog No. 2501M19) were quantified using commercially available enzyme-linked immunosorbent assay (ELISA) kits (Jiangsu Meimian Industry Co., Ltd., Yancheng, China) in strict accordance with the manufacturer’s instructions. Absorbance was measured at a wavelength of 450 nm immediately following the termination of the enzymatic reaction.

### 2.11. Statistical Analysis

Data are presented as mean ± standard deviation (SD) from a minimum of six independent experiments. Statistical significance was determined using GraphPad Prism software (v10.1.2) by applying one-way ANOVA with Tukey’s post hoc test for multiple groups or an unpaired Student’s *t*-test for two-group comparisons. Significance levels are denoted as follows: * *p* < 0.05, ** *p* < 0.01, *** *p* < 0.001; ns indicates not significant (*p* > 0.05).

## 3. Results and Discussion

### 3.1. Characterization

**Figure 1:**
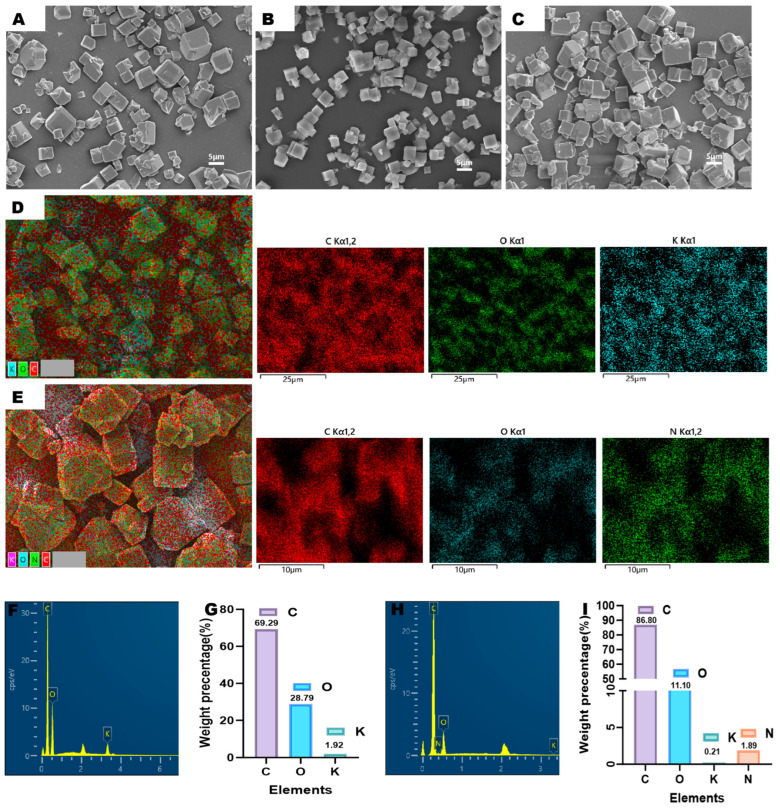
Scanning electron microscopy and elemental analysis. SEM image of COF (**A**), COF-FA (**B**), and FER@COF-FA (**C**). Scale bar = 5 μm. Elemental analysis of CD-MOF (**D**) and COF-FA (**E**). Graphical and quantitative elemental analysis of CD-MOF (**F**,**G**) and COF-FA (**H**,**I**).

The SEM imaging results confirm that both pristine COF particles and FA-modified COF exhibit uniform cubic morphology, as shown in Figure 1A,B. Critically, this structural integrity is preserved following sequential FA crosslinking and FER loading (Figure 1C), demonstrating that neither surface modification nor drug encapsulation compromises the framework integrity of the resulting FER@COF-FA delivery system. HPLC quantitative analysis results further substantiate successful functionalization; the FA surface grafting efficiency reached 9.98 ± 0.83%, confirming covalent modification of the COF carrier. Concurrently, FER loading achieved 14.97 ± 0.91%, validating effective drug encapsulation within the stabilized architecture (Figure S1).

The elemental profiles of CD-MOF and its functionalized derivative FER@COF-FA, revealed by EDS (Figure 1D–I), demonstrate profound compositional shifts that elucidate the structural modifications. CD-MOF’s ternary system, consisting of carbon (69.29%), oxygen (28.79%), and potassium (1.92%), reflects its γ-cyclodextrin (γ-CD) foundation, where K^+^ ions coordinate hydroxyl groups to stabilize the metal–organic framework (Figure 1F,G) [37,38]. Crucially, oxalyl chloride (ClCO-COCl) drives the transformation to COF by enabling two simultaneous processes: (1) covalent crosslinking between the numerous hydroxyl groups in γ-CD-MOF to form polyoxalate ester bonds; (2) elemental recomposition by substituting K^+^ ions [29]. Furthermore, following FA modification, the resultant COF-FA exhibits four elements: carbon (86.80%, +17.51%), nitrogen (1.89%, new), oxygen (11.10%, −17.69%), and trace potassium (0.21%, −1.71%) as illustrated in Figure 1H,I.

The carbon surge arises directly from oxalyl chloride’s high-carbon backbone (–CO–CO–), which inserts two carbon atoms per crosslink during ester bond formation (R–OH + ClCO–COCl → R–O–CO–CO–Cl + HCl). Concomitantly, FA’s pterin ring contributes additional carbon. The observed reduction in oxygen atomic percentage is attributed to compositional dilution resulting from the incorporation of these carbon-rich molecular components (OC and FA), coupled with the loss of potassium ions and associated oxygen-containing water molecules originally coordinated within the CD-MOF structure. It is important to emphasize that the oxygen atoms from the original hydroxyl groups are retained within the newly formed polyoxalate ester bonds and were not eliminated. The residual potassium (0.21%) confirms near-complete K^+^ displacement during crosslinking, while the emergence of nitrogen confirms successful FA integration.

Quantitatively, the data align with reaction stoichiometry, where (1) the 25.3% relative carbon increase (ΔC = 17.51%) exceeds FA’s carbon contribution, underscoring oxalyl chloride’s role. (2) Oxygen’s 61.5% decline (ΔO = 17.69%) correlates with consumed –OH groups. These shifts validate COF-FA as a covalently networked, organic-dominant framework that is distinct from CD-MOF’s ion-coordinated architecture. The EDS evidence, synergized with oxalyl chloride’s chemistry, thus establishes an unambiguous lineage from precursor to product.

**Figure 2:**
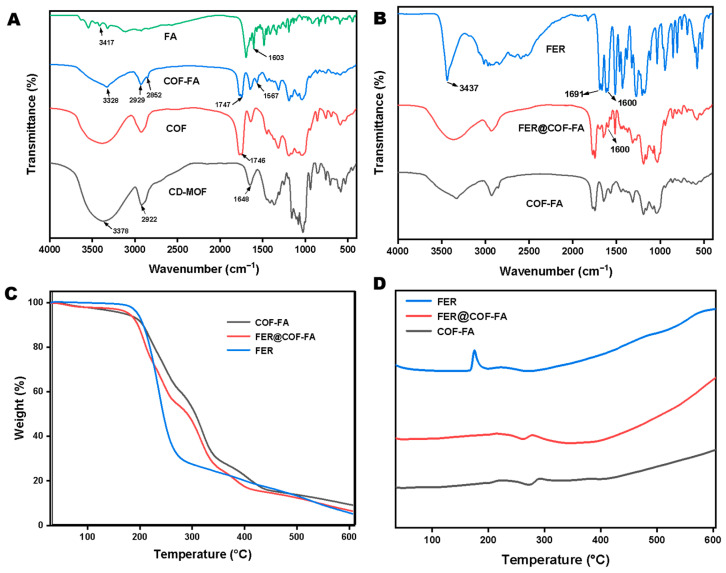
Characterization of CD-MOF, COF, and FER@COF-FA. FTIR analysis (**A**,**B**), Thermogravimetric analysis (**C**), and Differential Scanning Calorimetric analysis (**D**).

FT-IR analysis (Figure 2A,B) reveals critical structural distinctions between CD-MOF and COF, as summarized in Table S1. Briefly, CD-MOF displays a broad OH stretching vibration at 3378 cm^−1^, characteristic of O-H groups within γ-CD’s glucose rings, alongside a C-H stretching vibration at 2922 cm^−1^ attributed to CH_2_/CH groups, a feature conserved in both materials [39]. However, COF exhibits significant attenuation of the –OH peak, directly evidencing successful esterification between CD-MOF’s OH groups and OC. Further confirming this transformation, CD-MOF’s 1648 cm^−1^ peak (assigned to H-O-H bending of confined water) disappears in COF, while a new peak emerges at 1746 cm^−1^. This signal corresponds to C=O stretching of oxalate esters, verifying covalent crosslink formation via OC-mediated reactions.

Folic acid modification introduces distinct spectral signatures; its FT-IR spectrum shows a broad 3417 cm^−1^ peak from overlapping O-H (carboxyl/phenolic) and N-H (pterin ring) stretching vibrations, plus aromatic C=O stretching at 1603 cm^−1^ from p-aminobenzoyl/pterin moieties [40,41]. Following FA functionalization to yield COF-FA, new peaks materialize at 3328 cm^−1^ (N-H stretch) and 1567 cm^−1^ (aromatic C=C), confirming FA’s successful incorporation (Figure 2A). Critically, the preservation of COF’s oxalate carbonyl peak (1746 cm^−1^) alongside these new signals indicates that FA grafting occurs without disrupting the pre-established crosslinked framework (Figure 2B). Collectively, the spectral evolution diminished –OH vibrations, emergent oxalate carbonyls, and FA-specific N-H/aromatic bands, which further provides evidence for the sequential synthesis of (i) oxalyl chloride-driven crosslinking of CD-MOF into COF, and (ii) subsequent surface modification via FA to achieve COF-FA.

PXRD reveals the critical transition between the crystalline cubic CD-MOF into an amorphous cubic particle following introduction of the polyoxalate ester bond as shown by Figure S2. Meanwhile, TGA analysis reveals critical interactions between FER and the COF-FA carrier. As shown in Figure 2C and Figure S3, pure FER undergoes rapid mass loss between 180–277 °C, peaking at 237.98 °C. Following encapsulation within COF-FA, however, the FER@COF-FA exhibits fundamentally altered thermal behavior. Its decomposition profile aligns with the COF-FA carrier rather than free FER, with the maximum mass loss temperature shifting to 316.63 °C. This value closely approximates COF-FA’s decomposition peak (319.71 °C), indicating FER interacts strongly with the carrier matrix, as demonstrated in Figure 2C, confirming the successful incorporation rather than surface adsorption [42]. Furthermore, the DSC results (Figure 2D) provide complementary evidence to the TGA results. The pure FER displays a characteristic endothermic melting peak at 174.83 °C, this thermal signature disappears entirely in the FER@COF-FA thermogram across the 30–250 °C range [43]. The absence of FER’s melting transition confirms molecular-level dispersion and stabilization of the drug within the carrier framework. Collectively, these thermal transformations and the 78.65 °C upward shift in decomposition temperature and suppression of FER’s melting endotherm demonstrate successful encapsulation through non-covalent interactions that disrupt FER’s crystalline lattice while enhancing its thermal stability.

### 3.2. Evaluation of In Vitro H_2_O_2_ Scavenging Ability of COF-FA

**Figure 3:**
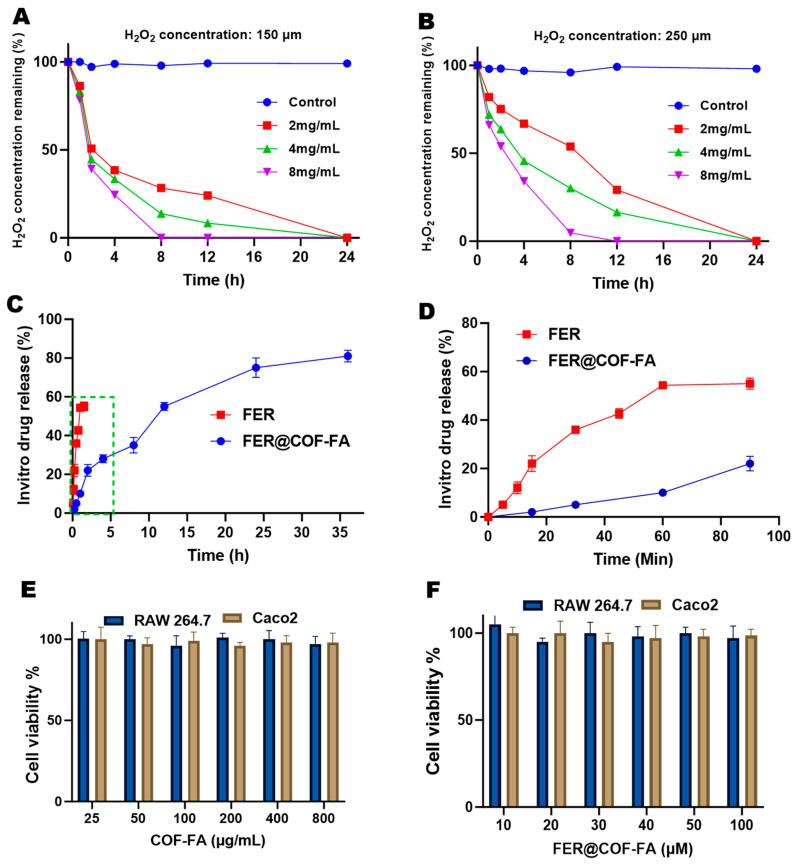
In vitro H_2_O_2_ scavenging ability of COF-FA in 150 μM H_2_O_2_ (**A**) & 250 μM H_2_O_2_ (**B**). In vitro drug release of Pure FER & FER@COF-FA (**C**), along with the inset, highlighted by a green dotted square, shows a zoomed view of the release behavior between 0 and 100 min (**D**). Cell viability study of COF-FA (**E**) & FER@COF-FA (**F**). Data are expressed as mean ± SD (n = 6).

The antioxidant capacity of COF particles was evaluated by measuring their ability to scavenge hydrogen peroxide (H_2_O_2_) and suppress intracellular reactive oxygen species (ROS). Mechanistically, it has been demonstrated that polyoxalate bonds in COF undergo hydrolysis in aqueous environments, producing oxalic acid that reacts stoichiometrically with H_2_O_2_ to form CO_2_ and water [28]. This reaction enables molar-equivalent H_2_O_2_ elimination per hydrolyzed unit. Experimental assessment at pH 7.4, H_2_O_2_ confirmed concentration- and time-dependent scavenging (Figure 3A,B). At 150 μM H_2_O_2_, COF-FA (2–8 mg/mL) progressively reduced residual peroxide over 4 h, with 8 mg/mL achieving 75.56% clearance (24.44 ± 0.69% residual) as shown in Figure 3A. When H_2_O_2_ concentration increased to 250 μM, the clearance trend persisted, but efficiency declined; 8 mg/mL COF-FA yielded 65.96% clearance (34.04 ± 1.93% residual). This 9.6% reduction in scavenging efficacy correlates directly with elevated peroxide levels, indicating substrate-limited reaction kinetics (Figure 3B). Critically, all tested concentrations demonstrated significant H_2_O_2_ removal, confirming COF-FA’s functionality as a peroxide-scavenging material across biologically relevant conditions.

### 3.3. FER In Vitro Release Result

The in vitro release profiles of FER and FER@COF-FA reveal distinct differences in their release behavior as demonstrated in Figure 3C,D. Free FER exhibited a rapid release, with most of the drug diffusing into the medium within 80 min. This burst release is characteristic of encapsulated drugs, reflecting the absence of a carrier system to control diffusion. Such a rapid release can lead to poor bioavailability and limited therapeutic efficiency, since the drug may be metabolized or cleared quickly from the body [44]. In contrast, FER@COF-FA demonstrated a much slower and sustained release profile (Figure 3D). The COF matrix effectively retained FER, preventing immediate diffusion and allowing a gradual release over an extended period (35 h). This controlled release behavior minimizes the burst effect observed in free FER and provides a more stable release pattern, which is advantageous for maintaining therapeutic concentrations over time.

It is important to note that this experiment was conducted under standard physiological conditions to establish a baseline sustained-release profile. While the design of the COF carrier, based on hydrolyzable polyoxalate esters, is intended for ROS-responsive drug release in the inflammatory microenvironment, a direct side-by-side comparison of the release kinetics in the presence and absence of high ROS concentrations (e.g., H_2_O_2_) was not performed in this study. Our claim of ROS-responsive potential is therefore strongly supported by the demonstrated ROS-scavenging capability of the carrier itself (Figure 3A,B), which confirms the occurrence of the key chemical reaction (hydrolysis of oxalate bonds by H_2_O_2_) that would trigger accelerated drug release. Future studies will explicitly measure release profiles under high-ROS conditions to provide direct kinetic evidence for this responsive behavior.

### 3.4. Cytotoxicity of COF-FA and FER@COF-FA Carrier

The cytotoxicity of COF, COF-FA, and FER@COF-FA was evaluated in RAW 264.7 macrophages and Caco-2 intestinal epithelial cells to assess their biocompatibility. For COF and COF-FA, a wide concentration range of 25–800 μg/mL was tested (Figure S4 and Figure 3E), while FER@COF-FA was examined at 10–100 μM based on the equivalent concentration of encapsulated FER (Figure 3F). Across all tested concentrations, both formulations maintained high cell viability, consistently above 90%, indicating negligible cytotoxicity toward either cell line.

In RAW 264.7 cells, which represent activated macrophages commonly associated with inflammation, COF-FA exhibited no significant dose-dependent reduction in viability. Similarly, FER@COF-FA preserved high cell survival, confirming that the encapsulation of FER did not increase toxicity. In Caco-2 cells, a model of intestinal epithelium relevant to ulcerative colitis, both COF-FA and FER@COF-FA again demonstrated excellent safety profiles, further supporting their compatibility for gastrointestinal applications. These results highlight the intrinsic biocompatibility of COF-FA as a carrier, which is essential for safe biomedical use. Moreover, the absence of additional toxicity upon FER loading confirms that FER@COF-FA retains the favorable safety characteristics of the carrier.

### 3.5. In Vivo Study

#### 3.5.1. Therapeutic Efficacy in DSS-Induced Colitis

**Figure 4:**
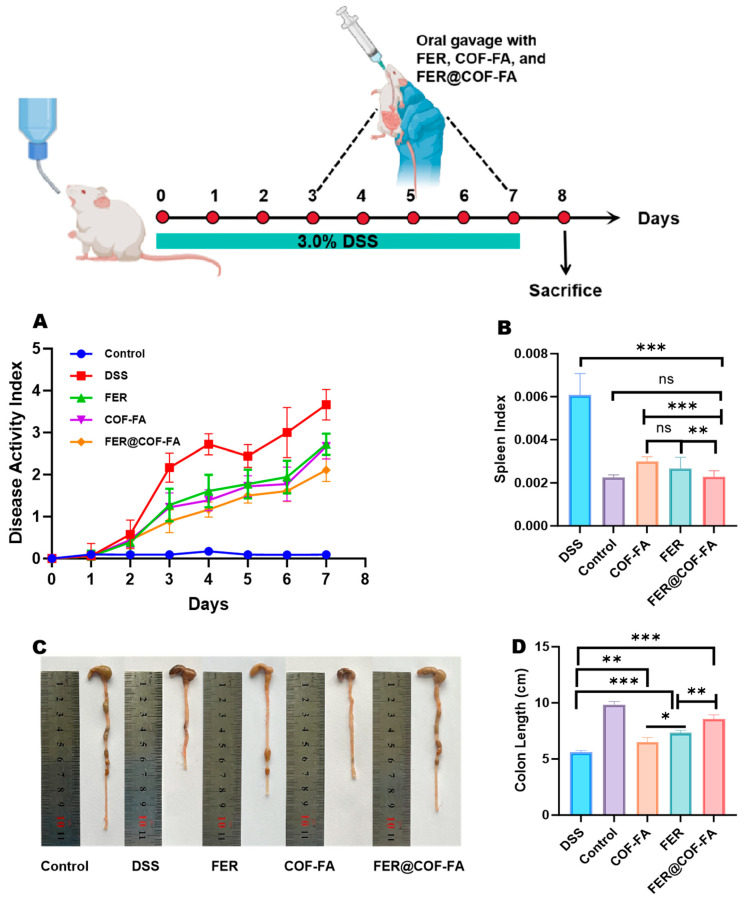
In vivo results: DAI score (**A**), Spleen index (**B**), Colon length of each group (**C**), Quantitative colon length analysis (**D**). Data are expressed as mean ± SD (n = 6). ns (not significant, *p* > 0.05), * (*p* < 0.05), ** (*p* < 0.01), *** (*p* < 0.001).

A UC model was established by administering 3% DSS ad libitum to mice. By day three, animals exhibited classic colitis symptoms, including weight loss, diarrhea, and hematochezia, while control mice remained clinically stable (Figure 4) [39]. Disease severity peaked at day seven, with the untreated DSS group showing maximal DAI scores reflecting progressive wasting and rectal bleeding. Therapeutic interventions demonstrated hierarchical efficacy, with FER significantly reducing DAI scores compared to the DSS group, confirming antioxidant-mediated symptom mitigation as shown in Figure 4A. The COF-FA carrier further attenuated disease severity beyond FER monotherapy, suggesting enhanced bioavailability through folic acid functionalization. Remarkably, FER@COF-FA particles achieved maximal clinical improvement, demonstrating synergistic alleviation of all symptoms.

Systemic and anatomical biomarkers provided quantitative validation of therapeutic outcomes. The spleen index, reflecting systemic inflammatory burden, increased significantly in DSS group mice compared to the control group (*p* < 0.001, Figure 4B). FER monotherapy reduced splenomegaly by 50.48%, indicating moderate immunomodulatory effects. COF-FA carrier alone achieved a 56.07% reduction (*p* < 0.001), surpassing free FER and highlighting the intrinsic anti-inflammatory properties of the functionalized delivery carrier. Notably, FER@COF-FA particles demonstrated maximal efficacy with a 62.35% decrease (*p* < 0.001), attributable to a synergistic effect combining anti-inflammatory/antioxidant delivery with likely folate receptor-mediated cellular uptake and enhanced permeability and retention (EPR) in the inflamed tissue. Several studies in cells and in vivo have demonstrated the efficacy of drug delivery systems targeting the folate receptor. This enhanced efficacy aligns with previous studies demonstrating the effectiveness of folate receptor-targeted drug delivery systems. Research has shown that fluorescently labeled nanoparticles exhibit enhanced uptake in folate receptor-expressing colon epithelial cells, activated macrophages, and inflamed colon tissue [13]. Additional studies have confirmed that folate-targeted dendrimers specifically bind to folate receptor-expressing macrophage cell lines in vitro and selectively accumulate in areas of inflammation in vivo [45]. Furthermore, modification with folic acid molecules has been shown to improve inflammatory cell targeting capability and ensure successful drug delivery to sites of inflammation [32,46,47,48]. Collectively, these findings provide a compelling rationale for the enhanced efficacy observed with the FER@COF-FA formulation.

Colon shortening, which is regarded as a hallmark of DSS-induced mucosal damage [23], reached 43.24% in the DSS group (*p* < 0.001, Figure 4C,D). While FER partially restored length (16.71%, *p* < 0.01), and COF-FA showed enhanced tissue protection (31.24%, *p* < 0.001). Interestingly, FER@COF-FA achieved near-complete restoration (50.10% improvement, *p* < 0.001), directly correlating with histopathological observations of preserved crypt architecture. This anatomical recovery signifies potent mitigation of fibrotic remodeling. Collectively, the hierarchical therapeutic efficacy (FER@COF-FA > COF-FA > FER) confirms three critical advantages: (1) folic acid functionalization enhances colonic retention through receptor-mediated binding; (2) COF-mediated antioxidant capability; (3) nanoencapsulation protects FER from degradation, enabling sustained ROS scavenging in inflamed tissues.

#### 3.5.2. Pathological Changes in the Colonic Tissue of Mice

**Figure 5:**
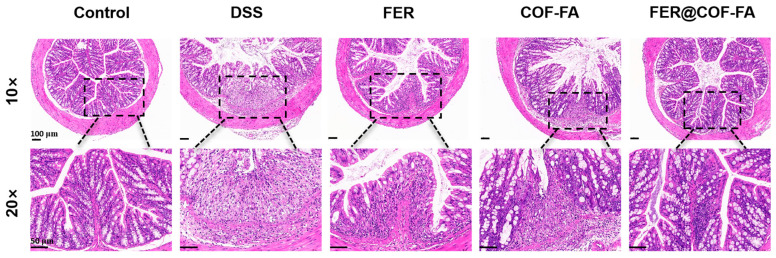
Representative microscopic images of colon HE histological sections from different treatment and model groups. Magnifications used: ×10 and ×20 (scale bars: 100 μm and 50 μm, respectively).

Histopathological analysis of H&E-stained colonic tissues revealed graded colitis severity across experimental groups (Figure 5). Control mice exhibited normal tissue architecture featuring regularly arranged mucosal epithelia, abundant goblet cells, absence of epithelial necrosis, and no interstitial congestion or inflammatory infiltration. Conversely, the DSS group showed severe structural disruption, including complete mucosal layer erosion, extensive ulceration with nuclear condensation and lysis, and transmural inflammatory cell infiltration [49]. Mice treated with free FER displayed mild architectural abnormalities characterized by intact epithelial organization but significantly diminished goblet cells and notable inflammation. The COF-FA group demonstrated moderate pathology with focal mucosal ulcers exhibiting nuclear degeneration, though goblet cell numbers remained normal amid substantial inflammatory infiltration. Critically, the FER@COF-FA group presented only minor deviations: preserved epithelial integrity without necrosis, abundant goblet cells, absence of interstitial congestion, and limited inflammatory cell infiltration.

These findings indicate that FER@COF-FA significantly outperforms FER in alleviating colonic damage, while COF-FA alone also confers therapeutic benefits. The superior histological preservation, particularly maintenance of epithelial integrity, goblet cell populations, and attenuated inflammation, confirms FER@COF-FA’s efficacy in mitigating DSS-induced colitis at the tissue level. This demonstrates substantial therapeutic potential.

#### 3.5.3. Effect of Inflammatory Factors in Mice Serum

**Figure 6:**
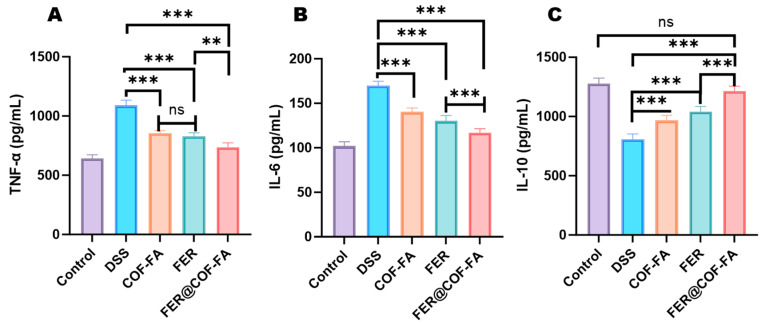
Comparison of the inflammatory markers TNF-α (**A**), IL-6 (**B**), and IL-10 (**C**). Data are expressed as mean ± SD (n = 6). ns (not significant, *p* > 0.05), ** (*p* < 0.01), *** (*p* < 0.001).

Quantitative analysis of serum cytokines revealed profound inflammatory dysregulation in experimental groups (Figure 6). Relative to controls, DSS group mice exhibited significantly elevated TNF-α and IL-6 concentrations (*p* < 0.001) alongside substantially reduced IL-10 (*p* < 0.001). Therapeutic interventions demonstrated progressive efficacy where the FER attenuated TNF-α by 23.92% and IL-6 by 23.25% while elevating IL-10 by 28.79% (all *p* < 0.001). COF-FA carrier monotherapy similarly modulated inflammatory mediators, reducing TNF-α (21.56%) and IL-6 (17.25%) while increasing IL-10 (19.52%, *p* < 0.001). Significantly, FER@COF-FA achieved maximal cytokine normalization, suppressing TNF-α (32.36%) and IL-6 (31.14%) while enhancing IL-10 (50.06%, all *p* < 0.001). This superior IL-10 induction significantly exceeded both comparator therapies, establishing FER@COF-FA as the most effective intervention for inflammatory mitigation. The amplified anti-inflammatory response confirms that nanoencapsulation potentiates FER’s therapeutic efficacy against colitis-associated tissue damage.

## 4. Conclusions

This study successfully developed FER@COF-FA, a multi-functional therapeutic platform for ulcerative colitis. The system synergistically combines a sustained-release covalent organic framework, intrinsic ROS-scavenging capability, and folate-mediated targeting potential. Comprehensive characterization validated the nanocarrier’s structure, antioxidant properties, and controlled release profile. In vivo evaluation demonstrated superior therapeutic efficacy compared to free ferulic acid, with significant improvements in disease activity, colon pathology, and the balance of inflammatory cytokines. The enhanced outcomes are consistent with a proposed mechanism involving site-specific delivery enhanced by ROS-responsive drug release and potential macrophage targeting.

Despite these promising results, this study has limitations that highlight directions for future research. The therapeutic efficacy was evaluated in a single animal model of DSS-induced colitis; subsequent studies employing additional models, such as TNBS-induced colitis, would strengthen the generalizability of our findings. Furthermore, while the superior performance of FER@COF-FA is highly suggestive of active targeting through folate receptor mediation, direct proof, such as cellular uptake studies in folate receptor-positive versus -negative cells or in vivo biodistribution imaging with a fluorescent tracer, was not obtained. Confirming this specific mechanism remains a key objective for future work. Further studies on long-term toxicity and biodistribution of COF-FA are essential for clinical translation.

However, the current findings establish FER@COF-FA as a promising targeted therapeutic strategy, providing a strong foundation for developing advanced nanoformulations to treat ulcerative colitis.

## Data Availability

All data generated or analyzed during this study are included in this published article and its supplementary information files. Further requests may be directed to the corresponding author.

## Supplementary Materials

The following supporting information can be downloaded at: https://www.mdpi.com/article/10.3390/pharmaceutics17101263/s1, Figure S1: Drug loading percent calculated before and after folic acid functionalization; Figure S2: Loss of crystallinity after oxalate crosslinking; Figure S3: DTG Analysis; Figure S4: Cell viability analysis of COF carrier after synthesis; Table S1. FTIR key vibrational frequencies for CD-MOF, COF, and COF-FA.
