# Supplementary material for: Folate-Functionalized ROS-Scavenging Covalent Organic Framework for Oral Targeted Delivery of Ferulic Acid in Ulcerative Colitis
Source: Pharmaceutics. 2025 Sep 26;17(10):1263. doi: 10.3390/pharmaceutics17101263 (PMC12566623; doi:10.3390/pharmaceutics17101263)
Supplement: Supplementary file 1 [file pharmaceutics-17-01263-s001.zip › pharmaceutics-3864575-supplementary.pdf]

Supplementary file

The standard curve of FER was drawn with mass concentration as the horizontal axis and absorbance as the vertical axis. The results showed that FER conformed to the Lambert-Beer law between the concentrations of 1.6 and 16 ug/mL, and had a good linear relationship. The regression equation was Y=0.1023X+0.0349; the correlation coefficient R2=0.9999. After calculation, the average FER loading was in FER@COF and FER@COF-FA is 22.02% ± 1.05% and 14.97% ± 0.91%.

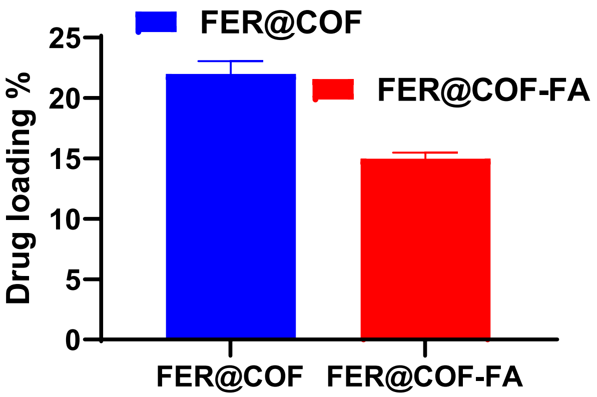

S1. Drug loading percent calculated before and after folic acid functionalization

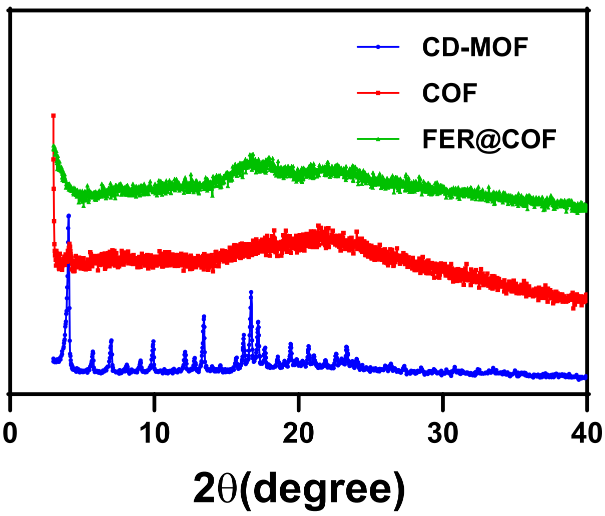

S2. Loss of crystalinity after oxalate crosslinking

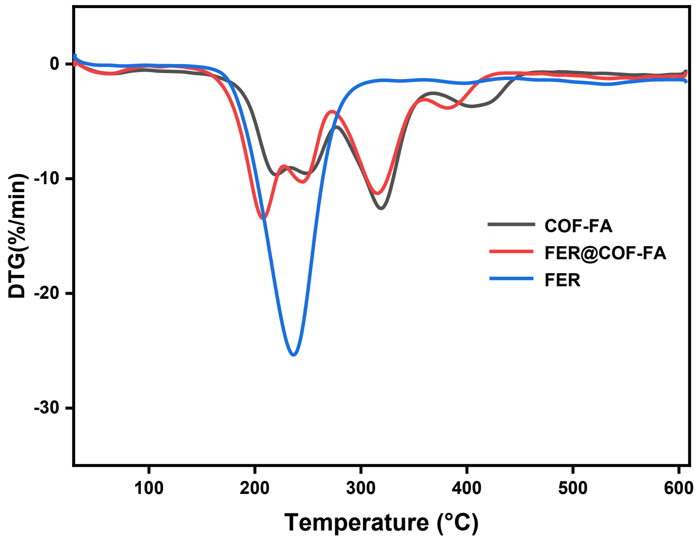

S3. DTG Analysis

Table S1. FTIR key vibrational frequencies for CD-MOF, COF, and COF-FA

Material	Wave number (cm⁻¹)	Assignment	Interpretation
CD-MOF	3378	ν(O-H) stretching of hydroxyl groups in γ-CD and coordinated water	Characteristic of the native metal-organic framework structure.
	2922	ν(C-H) asymmetric stretching of	Present in all carbon-based materials,

		CH_2/CH groups	confirming organic backbone.
	1648	δ(H-O-H) bending of confined water molecules	Indicates the presence of water within the CD-MOF pores.
COF	1746	ν(C=O) stretching of oxalate ester bonds (R-O-(C=O)-(C=O)-O-R')	New peak. Direct evidence of successful cross-linking via reaction between CD-MOF hydroxyls and oxalyl chloride.
	3378	ν(O-H) stretching	Significantly attenuated. Confirms consumption of hydroxyl groups during esterification.
	1648	δ(H-O-H) bending of water	Disappeared. Indicates removal of water and successful formation of a hydrophobic, cross-linked framework.
FA	3417	Broad band from overlapping ν(O-H) (carboxyl/phenolic) and ν(N-H) (pterin ring) stretching	Characteristic of folic acid structure.
	1603	ν(C=O) stretching of aromatic carbonyls (p-aminobenzoyl/pterin moieties)	Characteristic of FA structure
COF-FA	3328	ν(N-H) stretching	New peak. Confirms the successful incorporation of folic acid onto the COF surface.
	1567	ν(C=C) stretching of aromatic rings	New peak. Further confirms the presence of folic acid's aromatic structure.
	1746	ν(C=O) stretching of oxalate ester bonds	Preserved. Indicates that the folic acid functionalization did not disrupt the pre-formed covalent organic framework (COF) structure.

ν = stretching vibration; δ = bending vibration.

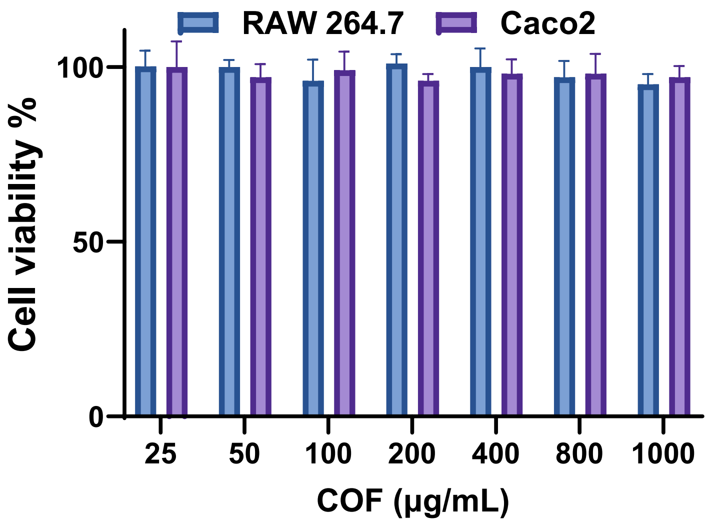

S4. Cell viability analysis of COF carrier after synthesis
